# Heat Transfer and Geometrical Analysis of Thermoelectric Converters Driven by Concentrated Solar Radiation

**DOI:** 10.3390/ma3042735

**Published:** 2010-04-14

**Authors:** Clemens Suter, Petr Tomeš, Anke Weidenkaff, Aldo Steinfeld

**Affiliations:** 1 Department of Mechanical and Process Engineering, ETH Zurich, 8092 Zurich, Switzerland; E-Mail: suterc@ethz.ch (C.S.); 2 Solid State Chemistry and Catalysis, Empa, 8600 Duebendorf, Switzerland; E-Mails: petr.tomes@empa.ch (P.T.); anke.weidenkaff@empa.ch (A.W.); 3 Solar Technology Laboratory, Paul Scherrer Institute, 5232 Villigen, Switzerland

**Keywords:** thermoelectricity, thermoelectric converter, solar, heat transfer, radiation, modeling, optimization

## Abstract

A heat transfer model that couples radiation/conduction/convection heat transfer with electrical potential distribution is developed for a thermoelectric converter (TEC) subjected to concentrated solar radiation. The 4-leg TEC module consists of two pairs of *p*-type La_1.98_Sr_0.02_CuO_4_ and *n*-type CaMn_0.98_Nb_0.02_O_3_ legs that are sandwiched between two ceramic Al_2_O_3_ hot/cold plates and connected electrically in series and thermally in parallel. The governing equations for heat transfer and electrical potential are formulated, discretized and solved numerically by applying the finite volume (FV) method. The model is validated in terms of experimentally measured temperatures and voltages/power using a set of TEC demonstrator modules, subjected to a peak radiative flux intensity of 300 suns. The heat transfer model is then applied to examine the effect of the geometrical parameters (e.g. length/width of legs) on the solar-to-electricity energy conversion efficiency.

## 1. Introduction

A thermoelectric converter (TEC) comprises *p*-type and *n*-type semiconductor legs sandwiched between two ceramic hot/cold plates and connected thermally in parallel and electrically in series [1,2,3]. The temperature gradient across the legs induces a voltage due to the Seebeck effect. The TEC performance is characterized by its figure-of-merit, ZT = S^2^T/(ρκ). Due to the relatively low heat-to-electricity conversion efficiencies approaching 5% for ZT ≤ 1, TECs have been mainly used in space applications. With the advent of novel functional ceramic materials, new high-temperature application areas are being considered, e.g. waste heat recovery and solar thermoelectric generation [4,5,6]. Previous heat transfer analyses considered compatibility factors [7,8,9] and heat conduction models [10,11]. In this paper, a FV-based heat transfer model of a TEC module is developed for simulating its thermal performance and analyzing the effect of the geometrical parameters. Coupled radiation/conduction/convection heat transfer with electrical potential distribution is considered for a TEC module directly exposed to concentrated solar radiation. The model is experimentally validated with measurements of temperature and voltages/power using a set of simplified 4-leg TEC modules that were directly irradiated. With these demonstrator (“proof-of-concept”) modules, the direct conversion of high-temperature solar heat is demonstrated [12]. However, no attempt has yet been undertaken to optimize the design or materials of these TEC modules for maximum energy conversion efficiency. Neither the designing of middle/large scale applications nor the combination with other technologies, e.g. PV cells, has been considered yet.

## 2. Experimental

**Figure 1:**
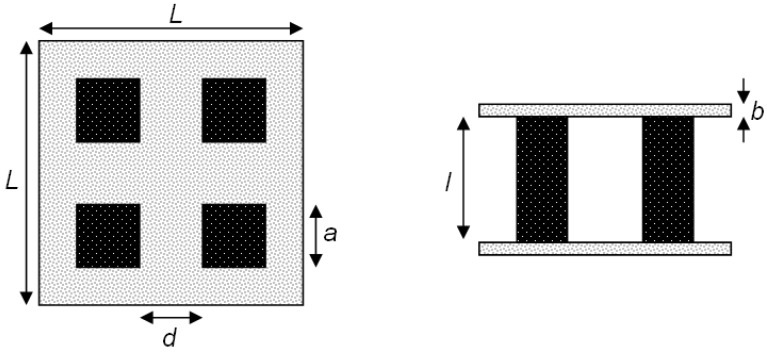
Top and front view of TEC module.

Figure 1 depicts a schematic of a 4-leg TEC module used in the experimental runs. Six demonstrator modules were fabricated with leg lengths *l* = 4, 5, and 10 mm (2 modules for each leg length). Each leg has a quadratic cross section of width *a* = 4.5 mm and a distance *d* = 10 mm from the neighboring leg. The *p*-type legs are made of La_1.98_Sr_0.02_CuO_4_; the *n*-type legs are made of CaMn_0.98_Nb_0.02_O_3_. These perovskite materials exhibit chemical and mechanical stability at high temperatures, but at the expense of having ZT ~ 0.05 [13]. The *L*x*L*x*b* = 30 × 30 × 0.25 mm absorber (hot) and cooling (cold) plates are made of Al_2_O_3_ with ~5% porosity. Additionally, the absorber plate is coated with graphite to augment its absorptivity.

**Figure 2:**
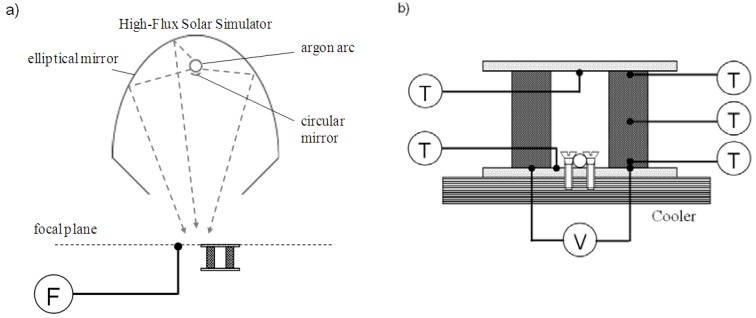
Schematic of the experimental setup at ETH’s High Flux Solar Simulator. (a) the TEC module is placed at HFSS’s focal plane; incident solar radiative fluxes measured by a thermogage (F). (b) position of type-K thermocouples (T) used to measure temperatures of the plates and of the hot end, middle, and cold end of the legs; terminals (V) provided at the cold ends for measuring the voltage/power output of the module. The cold plate was attached to a water-circuit cooler (denoted by screw fixation).

Experimentation was carried out at the ETH’s High Flux Solar Simulator (HFSS): a high-pressure Ar arc close-coupled to an elliptical reflector that delivers an external source of intense thermal radiation to simulate the heat transfer characteristics of highly concentrating solar systems [14]. The solar flux concentration is characterized by the mean concentration ratio $\tilde{C}$, defined as $\tilde{C}=Q_{\text{solar}}/\left(I\cdot \;A\right)$, where *Q*_solar_ is the solar power intercepted by a target of area *A*. The ratio $\tilde{C}$ is often expressed in units of “suns” when normalized to *I* = 1 kW/m^2^. The experimental set-up is shown schematically in Figures 2 (a) and (b). Incident radiative fluxes were measured by a thermogage (with an accuracy of ±3%) [15], placed symmetrically to the TEC module at the focal plane. The TEC was exposed to a maximum mean solar concentration ratio of 300 suns. K-type thermocouples (tip = 0.5 mm, spatial accuracy = ±0.25 mm) were used to measure temperatures of the plates and of the hot end, middle, and cold end of the legs. Terminals were provided at the cold ends for measuring the voltage/power output of the module. The cold plate was attached to a water-circuit cooler at room temperature.

**Figure 3:**
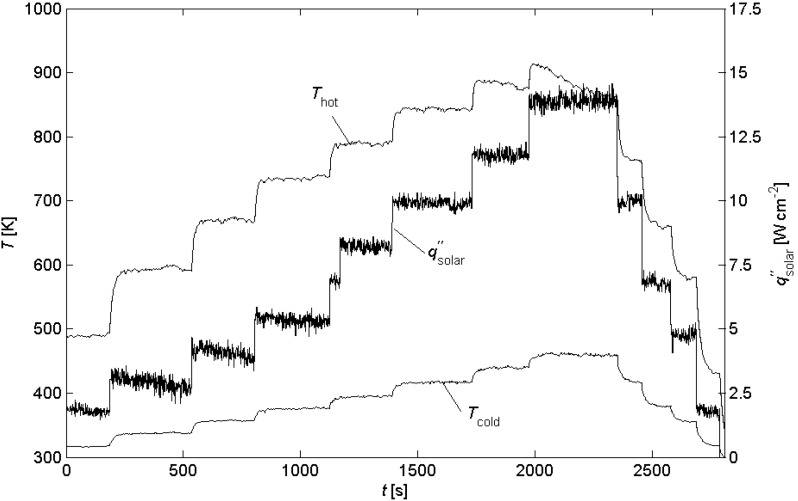
Temperature of hot and cold plates and solar radiative flux as a function of time during a representative experimental run for module with *l* = 4 mm.

**Figure 4:**
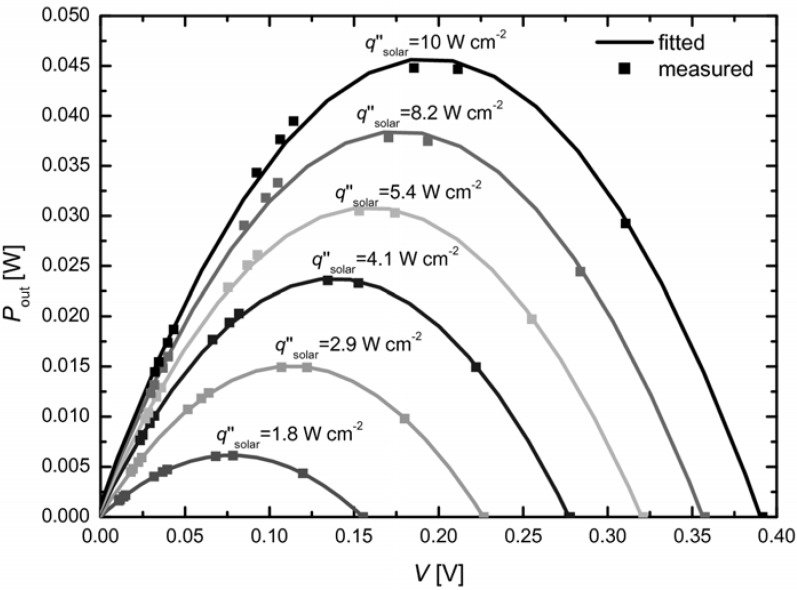
Fitted and measured voltage-power curves for incident solar radiative fluxes in the range $q_{\text{solar}}^{\prime \prime }$ = 1.8 – 10 W cm^-2^, and for external loads with resistance in the range *R*_load_ = 0.1-3.5 Ω for module with *l* = 4 mm.

The temperature and solar radiative flux as a function of time are shown in Figure 3 for a representative experimental run using a module with leg length *l* = 4 mm. The incident solar radiation was increased stepwise and held at constant level for 3-5-minute intervals. Due to the low thermal inertia and fast temperature response, steady-state conditions are assumed for each time interval. Maximum temperature was 625°C, at which graphite is no longer stable and starts to burn. For the same module (*l* = 4 mm), Figure 4 shows the theoretical and measured voltage-power curves for incident solar radiative fluxes in the range $q_{\text{solar}}^{\prime \prime }$ = 1.8–10 W cm^-2^, and for external loads with resistance in the range *R*_load_ = 0.1-3.5 Ω. A parabola, which corresponds to the ideal voltage source with an internal resistance [16], is fitted through the data points. The maximum power is *P*_max_ = 0.006, 0.015, 0.023, 0.031, 0.038 and 0.046 W for $q_{\text{solar}}^{\prime \prime }$ = 1.8, 2.9, 4.1, 5.4, 8.2 and 10 W cm^-2^, respectively.

**Figure 5:**
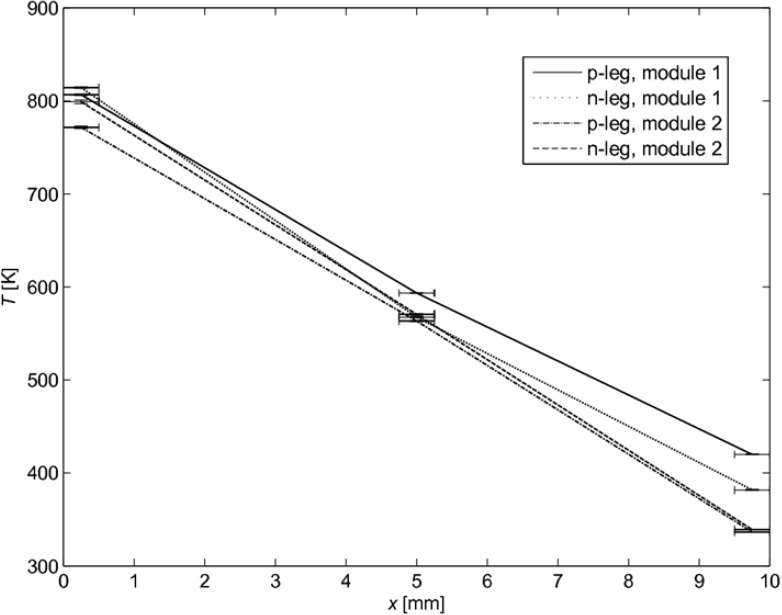
Temperature distribution along the p- and n-type legs for two modules with *l* = 10 mm. Error bars indicate spatial accuracy (±0.25 mm) of thermocouple placing.

The measured temperature distribution for two tested modules with leg length *l* = 10 mm is shown in Figure 5 for $q_{\text{solar}}^{\prime \prime }$ = 6 W cm^-2^. As expected, the quasi linear profile indicates a predominant heat transfer by conduction across the legs. The abnormal behavior of 100 K temperature difference at the cold side is presumably due to the incorporation of the screw fixation (see Figure 2 (b)) causing different heat transfer rates.

**Figure 6:**
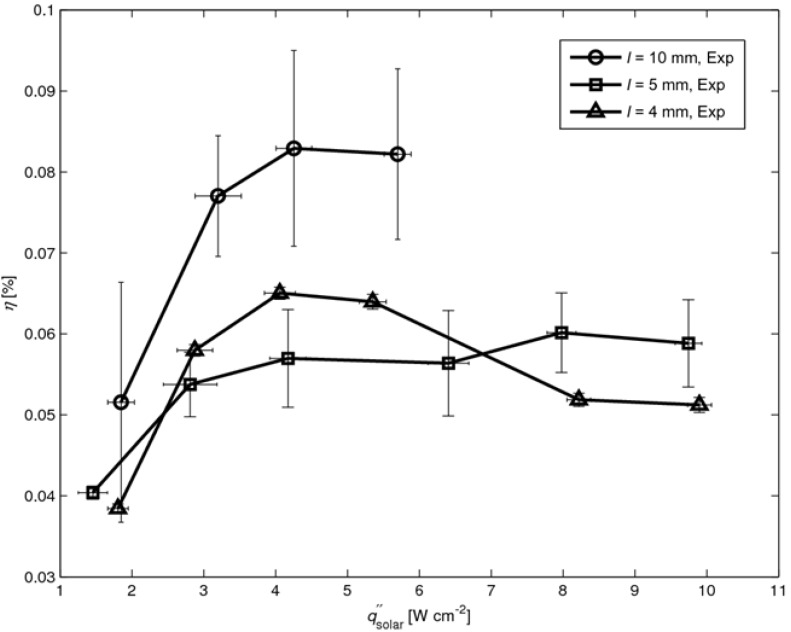
Efficiency *η* as a function of solar radiative flux for three modules with *l* = 4, 5, and 10 mm. Error bars indicating uncertainty of incident solar radiative flux and efficiency due to uncertain contact resistance.

*Efficiency* ― The solar-to-power efficiency of the TEC module is defined as:

(1) $$\eta =\frac{P_{\max}}{A_{abs}\cdot {q\prime \prime }_{solar}}$$

where *P*_max_ is the maximal power output and $q_{\text{solar}}^{\prime \prime }$ the mean solar radiative flux incident over the absorber surface *A*_absorber_. For modules with leg lengths *l* = 4, 5, and 10 mm the maximal power outputs *P*_max_ are 45.6, 51.6 and 42.2 mW for $q_{\text{solar}}^{\prime \prime }$ = 9.9, 9.7, and 5.7 W cm^-1^, respectively. The efficiency *η* as a function of solar radiative flux is shown in Figure 6 for *l* = 4, 5, and 10 mm. The curves are plotted up to the maximal solar flux of 9.9, 9.7 and 5.7 W cm^-1^, respectively, for which *T*_hot_ = 625°C is reached. Higher solar fluxes resulted in the burning of the graphite coating. The efficiency increases with $q_{\text{solar}}^{\prime \prime }$ as a result of the higher temperature difference across the legs, which in turn corresponds to a higher Carnot limitation [11]. In contrast, *η* decreases with *T* as re-radiation losses are proportional to *T*^4^. Thus, an optimum $q_{\text{solar}}^{\prime \prime }$ for maximum *η* is expected. For *l* = 4 mm, *η*_max_ = 0.065% at $q_{\text{solar}}^{\prime \prime }$ = 4 W cm^-2^. For *l* = 5 mm *η*_max_ = 0.06% at $q_{\text{solar}}^{\prime \prime }$ = 8 W cm^-2^. For *l* = 10 mm, *η*_max_ = 0.083% at $q_{\text{solar}}^{\prime \prime }$ = 4 W cm^-2^.

## 3. Heat Transfer Model

**Figure 7:**
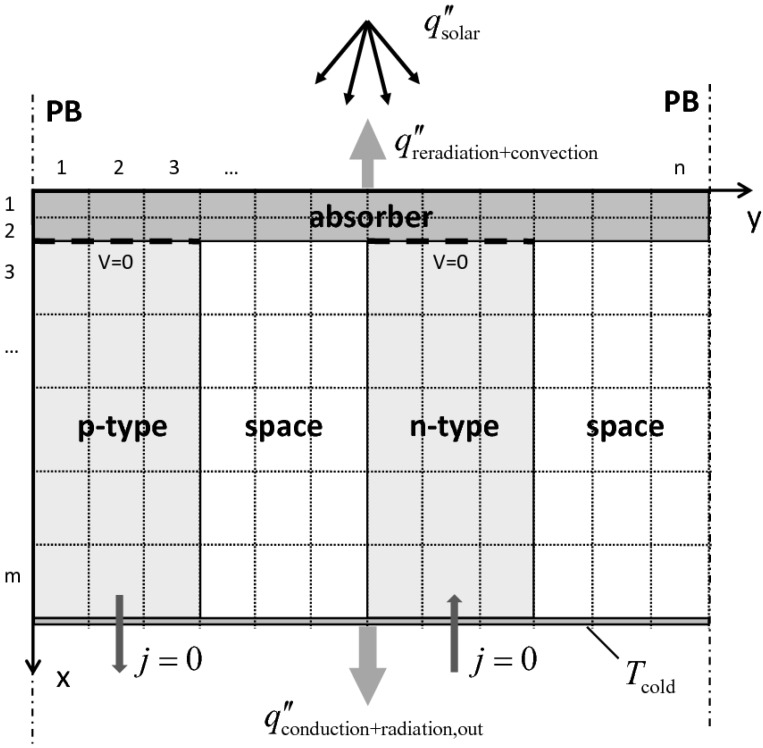
Scheme of the model domain (divided into m × n cells). Indicated are the boundary conditions.

A 2D steady-state heat transfer model is formulated. A cross section of the model domain, divided into *m* × *n* cells, is depicted in Figure 7. It contains the three major components: the absorber plate, one p- and one n-leg (P/N), and the space in-between. The domain is assumed to be infinitely long; therefore, periodic boundaries are set at the sides. The heat transfer modes considered are: (1) conduction in the complete domain, and (2) radiative heat transfer among all surfaces for two approaches: (a) assuming a semi-transparent absorber plate; (b) assuming an opaque absorber plate. It is further assumed: (*i*) the p/n solids are opaque, gray and diffuse scattering; (*ii*) gas phase is radiatively non-participating and its refractive index is equal to unity; (*iiia*) the absorber plate is radiatively participating with isotropic scattering and with temperature and wavelength independent extinction coefficient *β*_abs_ and albedo *ω*_abs_; (*iiib*) the absorber plate is opaque, gray and diffuse scattering; (*iv*) convection is only considered from top of the hot plate; (*v*) open circuit voltage (*j* = 0). *P*_max_ and *η* are calculated based on the matched load assumption, given by:

(2) $$P_{\max}=\frac{1}{4}\frac{V_{\text{OC}}^{2}}{R_{\text{internal}}+R_{\text{contact}}}$$

where *V*_OC_ is the open circuit voltage, *R*_internal_ the internal resistance of the TEC module, and *R*_contact_ the contact resistance between legs and conduction strips.

*Conservation equations* ― The steady-state energy conservation equation applied to the absorber for approach (a) is given by:

(3) $$\nabla \left(\kappa_{\text{absorber}}\cdot \nabla T\right)-q_{\text{radiation}}^{\prime \prime \prime }=0$$

where *k*_absorber_ is the absorber thermal conductivity and $q_{\text{radiation}}^{\prime \prime \prime }$ is the radiative volumetric heat source. For approach (b), $q_{\text{radiation}}^{\prime \prime \prime }$ = 0 and an additional boundary condition is necessary (see subchapter “boundary conditions”). The steady-state conservation equations applied to the legs (P/N domain) are given by:

(4) $$\text{Energy: }\nabla \left(\kappa_{\text{leg}}\cdot \nabla T\right)+\underset{j=0}{\underset{︸}{\rho_{\text{leg}}\cdot j^{2}}}-\underset{j=0}{\underset{︸}{T\cdot \frac{dS_{\text{leg}}}{dT}\cdot j\cdot \nabla T}}=0$$

with thermal conductivity *k*_leg_, electrical resistivity *ρ*_leg_, current per area *j*, and Seebeck coefficient *S*_leg_. Note that the two current terms cancel due to the open circuit condition:

(5) $$\text{Current: }\nabla j=0\text{, where }j=-{\frac{1}{\rho }}_{\text{leg}}\left[\nabla \left(\underset{const}{\underset{︸}{\frac{\mu_{\text{leg}}}{e_{\text{leg}}}}}+V\right)+S_{\text{leg}}\cdot \nabla T\right]$$

with electrical resistivity *ρ*_leg_, chemical potential *μ*_leg_, and the charge of charged particles of current *e*_leg_ and Seebeck coefficient *S*_leg_ [11]. Note that the gradient of $\frac{\mu_{\text{leg}}}{e_{\text{leg}}}$ cancels as this term is assumed constant.

For approach (a), the radiative heat transfer within the absorber plate is determined by the collision-based Monte Carlo (MC) method [17]. The radiative source term $q_{\text{radiation}}^{\prime \prime \prime }$ is approximated by:

(6) $$q_{\text{radiation}}^{\prime \prime \prime }\approx \frac{N_{\text{absorbed}}\cdot q_{\text{ray}}}{\Delta V}-2\beta_{\text{absorber}}\left(1-\omega_{\text{absorber}}\right)\sigma T^{4}$$

where *q*_ray_ is the power carried by a single ray, *N*_absorbed_ the number of rays absorbed within a control volume *∆V*, *β*_absorber_ the extinction coefficient, and *ω*_absorber_ its albedo. Thus, the net radiative flux $q_{\text{radiation,net}}^{\prime \prime }$ of the surfaces is calculated as:

(7) $$q_{\text{radiation,net}}^{\prime \prime }\approx \frac{N_{\text{absorbed}}\cdot q_{\text{ray}}}{\Delta A}-\varepsilon_{\text{surface}}\sigma T^{4}$$

where *q*_ray_ is the power carried by a single ray, *N*_absorbed_ the number of rays absorbed within a control surface *∆A*, and ε_surface_ its emissivity. For approach (b), the net radiative flux $q_{\text{radiation,net}}^{\prime \prime }$ from inner surface elements is computed using the radiosity method (enclosure theory) [18], assuming p/n-type surfaces with emissivity ε_P_, ε_N_, respectively, and uncoated (white) surfaces from absorber and cold plates with emissivity ε_absorber_. The corresponding system of equations is given by [17]:

(8) $$\begin{array}{l} \sum_{j=1}^{2(m_{\text{P/N}}+n_{\text{space}})}\left(\frac{\delta_{kj}}{\varepsilon_{j}}-F_{k-j}\frac{1-\varepsilon_{j}}{\varepsilon_{j}}\right)q_{\text{radiation,net,j}}^{\prime \prime }=\sum_{j=1}^{2(m_{\text{P/N}}+n_{\text{space}})}\left(\delta_{kj}-F_{k-j}\right)\sigma T_{j}^{4}\\ \text{ for }k=1...2(m_{\text{P/N}}+n_{\text{space}}) \end{array}$$

where *δ* is the Kronecker function, *m*_P/N_ is the number of p/n-type elements in x-direction and *n*_space_ the number of elements in y-direction. The view factors *F_k-j_* are calculated by applying reciprocity relations (*A*_1_*F*_1-2_=*A*_2_*F*_2-1_), enclosure criterion ($\sum_{j=1}^{N}F_{k-j}=1$), and tabulated view factors [19].

For simplicity, 2D geometry is considered. As the total absorber surface per leg must be the same for 3D and 2D geometries, the distance *d* between the legs for 3D is transformed to d* for 2D. Similarly, the thermal conductivity in the direction along the plate is as adjusted as $k_{\text{absorber}}^{*}$.

*Boundary conditions* ― $q_{\text{solar}}^{\prime \prime }$ from the HFSS is assumed to be uniformly distributed. The heat losses from the absorber’s top include re-radiation and free convection. Re-radiation is calculated by MC for approach (a), and by introducing a new boundary condition for approach (b): $q_{\text{reradiation}}^{\prime \prime }=\varepsilon_{\text{absorber,coated}}\sigma T^{4}$. Free convection $q_{\text{convection}}^{\prime \prime }$ to the environment is calculated using a Nusselt correlation for a horizontal flat plate [20]:

(9) $$\begin{array}{l} {\text{Nu}}_{L}=0.54\cdot {\text{Ra}}_{L}^{1/4}\;\left(10^{4}\leq Ra_{L}\leq 10^{7}\right)\\ {\text{Ra}}_{L}={\text{Gr}}_{L}\cdot \mathrm{Pr}=\frac{g\beta \left(T_{surface}-T_{\infty }\right)L^{3}}{\nu \alpha } \end{array}$$

where Nu, Ra, Gr, and Pr are the Nusselt, Rayleigh, Grashof, and Prandtl numbers, *g* the gravitational acceleration, *β* the volumetric thermal expansion coefficient, ν the kinematic viscosity, α the thermal diffusity, *T*_surface_ the surface temperature, *T*_∞_ the surroundings temperature, and *L* the characteristic length (here the absorber width, *L* = 30 mm). The outgoing heat flux contains radiation losses $q_{\text{radiation,out}}^{\prime \prime }$ through the space to the cold plate as well as conduction losses $q_{\text{conduction}}^{\prime \prime }$ through the legs to the cold plate. $q_{radiation,out}^{\prime \prime }$ is either calculated by MC for approach (a), and by the radiosity method for approach (b).

*Numerical solution* ― The finite volume technique (FV) is applied to discretize the governing equations (3) and (4) and solve the PDE system iteratively using the successive over-relaxation (SOR) method [21] implemented in FORTRAN. The algorithm is repeated until the convergence criterion:

(10) $$\left|\frac{T_{i,j}^{\gamma }-T_{i,j}^{\gamma -1}}{T_{i,j}^{\gamma }}\right|\leq \varepsilon$$

for all elements *i*,*j* after γ iterations is satisfied, with ε < 10^-6^ and the overall energy balance satisfied within 0.1%. After convergence, the potential distribution is calculated. A convergence study indicated optimal trade-off between accuracy and computational time with a grid containing 425 elements.

**Figure 8:**
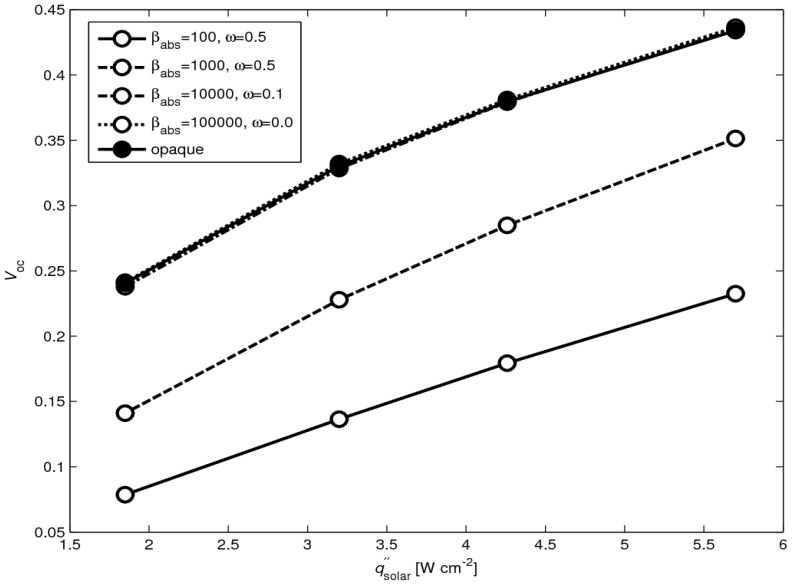
Simulated *V*_OC_ as a function of solar radiative flux for *l* = 10 mm for approach (a) with *β*_absorber_ = 100, *ω* = 0.5; *β*_absorber_ = 1000, *ω* = 0.5; *β*_absorber_ = 10000, *ω* = 0.1; *β*_absorber_ = 100000, *ω* = 0.0 and for opaque approach (b).

The difference between the *V*_OC_ calculated by the two approaches (a) and (b) for analyzing the radiative heat transfer is shown in Figure 8 for *l* = 10 mm. Different radiation properties (*β*_abs_, *ω*) of the absorber plate have been tested. For *β*_abs_➔∞ and *ω*➔0, no incoming radiation is transmitted through the absorber, and the solution obtained by approach (a) moves toward that for an opaque absorber obtained by approach (b). Since the absorber plate used in the measurements can be well approximated by an opaque surface, only approach (b) is applied in the analysis that follows.

## 4. Model Validation

Validation is accomplished for the open circuit voltage *V*_oc_, as this value is the most reliable magnitude to measure and is directly proportional to the mean temperature difference across the legs. The baseline parameter used for the model simulations are listed in Table 1.

**Table 1:** Baseline parameters.

Parameter	Value	Unit	Source
*l*	4 - 15	mm	measured/varied
*a*	3 - 6	mm	measured/varied
*d*	1 - 10	mm	measured/varied
*ε*_absorber,coated_	0.95	-	[22]
*ε*_absorber_	0.3	-	[22]
*ε*_P/N_	0.7	-	assumed
*β*_absorber_	100 - 100000	m^-1^	varied
*ω*	0.0 - 0.5	-	varied
k^*^_absorber_	250	W m^-1^ K^-1^	assumed
*k*_absorber_	1.78 × 10^-11^*T*^4^–9.79 × 10^-8^*T^3^*+2.02 × 10^-4^*T^2^*–1.90 × 10^-1^*T*+75.77	W m^-1^ K^-1^	[23]
*k*_P/N_	~ 1 – 2.5/~ 1.75 – 3	W m^-1^ K^-1^	measured
*S*_P/N_	~ 120 – 260/~ -170 – -230	μV K^-1^	measured
*ρ*_P/N_	~ 0.025 - 0.05/~ 0.02 – 0.036	Ω cm	measured
*T*_∞_	300	K	assumed
*T*_cold_	300	K	assumed
*R*_contact_	0.40-0.66	Ω	[12]/assumed

**Figure 9:**
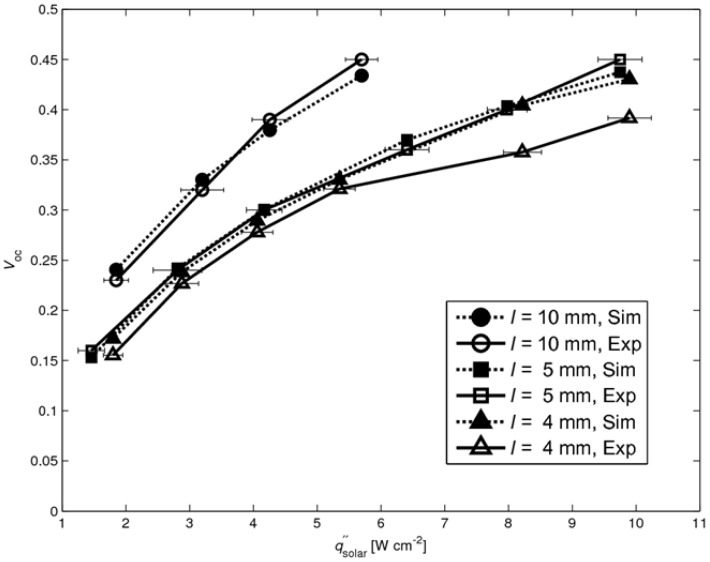
Simulated and experimental *V*_OC_ as a function of solar radiative flux for *l* = 4, 5, 10 mm. Error bars indicating uncertainty of incident solar radiative flux.

The experimentally measured and numerically calculated *V*_OC_ are shown in Figure 9 for *l* = 4, 5, and 10 mm. A reasonable good agreement is observed, except for the 4 mm case at high fluxes ($q_{solar}^{\prime \prime }$ > 8 W cm^-2^), where the model predicts a 15% higher value. This discrepancy is attributed to the insufficient cooling of the cold plate at high fluxes, as evidenced by a rise of its temperature, which in turn caused higher absorber plate temperature and, consequently, higher re-radiation losses. Thus, the temperature difference across the legs is shifted to higher temperatures and reduced due to the higher re-radiation.

**Figure 10:**
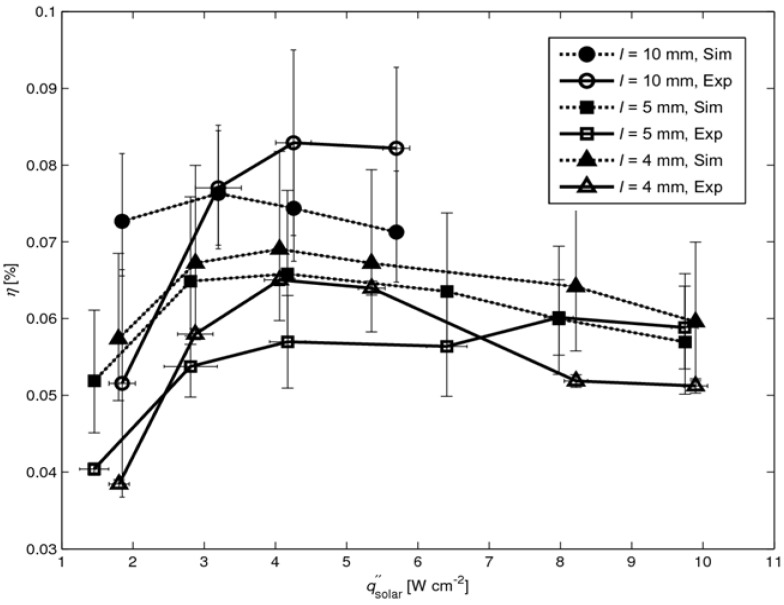
Simulated and experimental *η* as a function of solar radiative flux for *l* = 4, 5, 10 mm (experimental data from Figure 6). Error bars for simulated data points indicating uncertainty of contact resistance.

The numerically simulated solar-to-power efficiencies are shown in Figure 10 (together with the experimentally determined efficiencies from Figure 6), calculated using Equation (1) with maximal power output *P*_max_ from Equation (2). The internal leg resistances *R*_internal_ are calculated according to:

(11) $$R_{\text{internal}}=\sum_{i}\rho_{\text{leg,i}}\cdot \frac{\Delta x}{a^{2}}$$

where *ρ*_leg,i_ is the leg’s temperature dependent electrical resistivity, *Δx* the cell length in x-direction, *a* the width of the leg and *i* the index of summation over the number of cells in x-direction along the leg (see Figure 7). The mean contact resistance is assumed to be 0.53 ± 0.13 Ω for all cases, determined in [12]. The calculated values lie in the same range as the measured ones, expect for the 4 mm case at high fluxes ($q_{\text{solar}}^{\prime \prime }$ >8 W cm^-2^) which result from the overestimated *V*_OC_ (see Figure 9).

**Figure 11:**
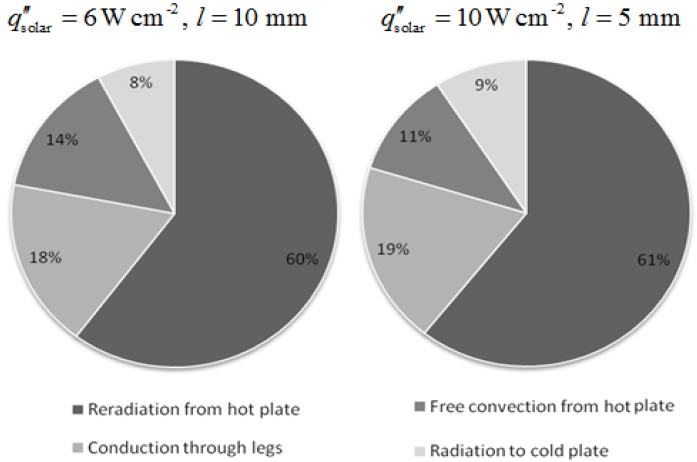
Percentage of ***Q*_solar_** transferred by the heat transfer modes.

The percentage of *Q*_solar_ transferred by the different heat transfer modes is shown in Figure 11 for two cases; 1) $q_{\text{solar}}^{\prime \prime }$ = 6 W cm^-2^ and *l* = 10 mm leg length, and 2) $q_{\text{solar}}^{\prime \prime }$ = 10 W m^-2^ and *l* = 5 mm. In both cases, the heat losses by re-radiation and free convection from the absorber plate represent more than 70% of *Q*_solar_. About 20% of *Q*_solar_ is transferred by conduction through the legs, and <10% is lost by radiation to the cold plate.

**Figure 12:**
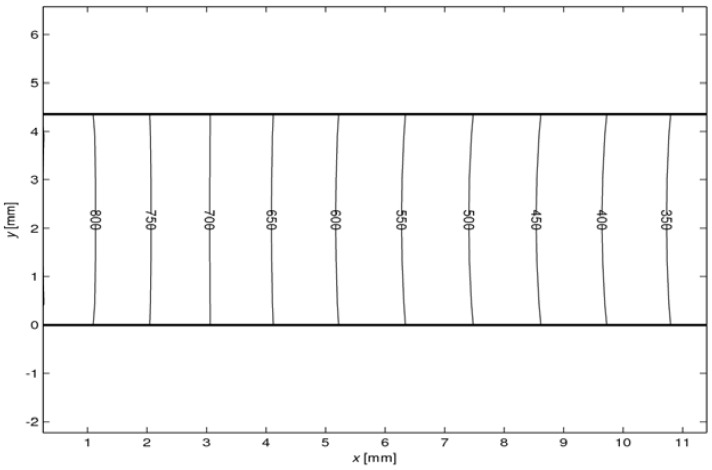
2D temperature profile in p-type leg for *l* = 10 mm at $q_{\text{solar}}^{\prime \prime }$ = 6 W m^-2^.

Figure 12 shows the temperature distribution along a p-type leg of *l* = 10 mm obtained for $q_{\text{solar}}^{\prime \prime }$ = 6 W m^-2^. A comparable distribution is obtained for an n-type leg. The profile is linear, as corroborated by the experimental data (see Figure 5). Perpendicular to the length axis, the temperature is almost uniform, with a slightly higher temperature at the surface because of the radiative exchange among legs and plates. The small temperature gradient indicates that this radiative heat exchange is not predominant, as confirmed by the fact that <10% of *Q*_solar_ is lost by radiation to the cold plate (see Figure 11).

## 5. Efficiency

*Leg length* *―*The simulated dimensions of the modules are *l* = 5 - 15 mm, *a* = 4.5 mm, and plates with *L*x*L*x*b* = 30 × 30 × 0.25 mm. The cold plate temperature is set to 300 K. The contact resistance is *R*_Contact_ = 0.55 Ω. The solar radiative fluxes are varied in the range $q_{\text{solar}}^{\prime \prime }={\text{2 - 10 W cm}}^{-2}$. The black coating of the absorber is assumed stable for all temperatures. The baseline parameter used for the model simulations are listed in Table 1.

**Figure 13:**
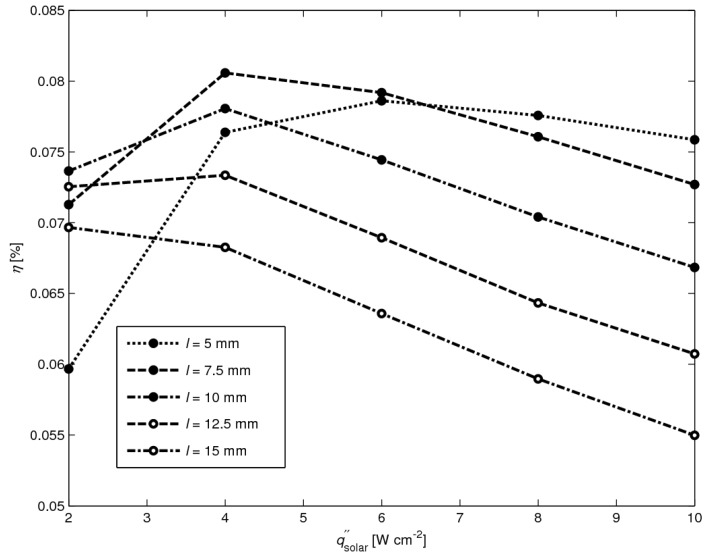
Efficiency as a function of solar radiative flux for *l* = 5 mm, *l* = 7.5 mm, *l* = 10 mm, *l* = 12.5 mm and *l* = 15 mm.

Figure 13 shows the efficiency as a function of solar radiative flux for *l* = 5, 7.5, 10, 12.5 and 15 mm. The highest efficiency *η* = 0.081% is obtained for *l* = 7.5 mm at $q_{\text{solar}}^{\prime \prime }$ = 4 W cm^-2^. Note that *l* = 7.5 mm is not optimal in the whole solar radiative flux range. For $q_{\text{solar}}^{\prime \prime }$ < 3 W cm^-2^, *l* = 10 mm is most efficient, whereas for $q_{\text{solar}}^{\prime \prime }$ > 7 W cm^-2^, *l* = 5 mm is most efficient. Thus, for increasing solar radiative fluxes, the optimal leg length decreases.

**Figure 14:**
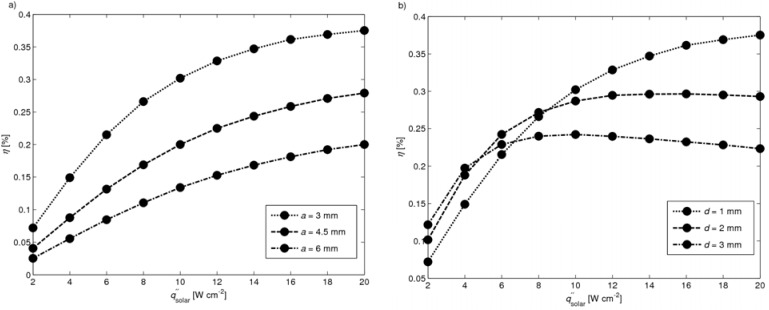
Efficiency as a function of solar radiative flux with *l* = 7.5 mm for **(a)** *d* = 1 mm and *a* = 3, 4.5, 6 mm and for **(b)** *a* = 3 mm and *d* = 1, 2, 3 mm.

*Leg width and distance between neighboring legs* *―* For practical manufacturing purposes, it is assumed that the minimal leg width is *a* = 3 mm and the minimal distance *d* = 1 mm. In Figure 14, the efficiencies are plotted as a function of solar radiative flux in the range $q_{\text{solar}}^{\prime \prime }$ = 2 – 20 W cm^-1^ for a module with a leg length *l* = 7.5 mm and for: (a) *d* = 1 mm and *a* = 3, 4.5, 6 mm, and (b) *a* = 3 mm and *d* = 1, 2, 3 mm. Highest efficiencies are obtained for *a* = 3 mm in the whole solar flux range, and for *d* = 1 mm in the range $q_{\text{solar}}^{\prime \prime }$ = 8 – 20 W cm^-2^. The peak *η* = 0.375% at $q_{\text{solar}}^{\prime \prime }$ = 20 W cm^-2^ is obtained for *a* = 3 mm and *d* = 1 mm, *i.e.* for the smallest leg width and distance between neighboring legs considered.

## 6. Summary and Conclusions

The conversion of high-temperature solar heat was demonstrated using a set of non-optimized TEC modules exposed to concentrated solar radiation. A 2D heat transfer numerical model of a TEC module has been implemented and validated based on experimental data. Two different approaches of modeling the radiation have been applied, namely the Monte Carlo method considering a semi-transparent absorber plate and the radiosity method considering only opaque surfaces. The absorber plate used in the measurements can be well approximated by an opaque surface. The heat transfer analysis for 4-leg modules with leg length *l* = 5–10 mm and absorber plates of *L* × *L* × *b* = 30 × 30 × 0.25 mm indicated that more than 70% of the incident solar power is lost due to re-radiation and free convection from the absorber, while 20% is conducted through the legs and <10% is lost by radiation to the cold plate. Heat conduction is the predominant mode of heat transfer across the legs, as corroborated by the measured and simulated linear temperature profiles across the legs. The optimal leg length of the 4-leg module with leg width *a* = 4.5 mm and plates *L* × *L* × *b* = 30 × 30 × 0.25 mm is *l* = 7.5 mm, which results in a maximal efficiency of 0.081% at $q_{\text{solar}}^{\prime \prime }$ = 4 W cm^-2^. Smaller leg width and distance between neighboring legs, namely, width *a* = 3 mm and gap *d* = 1 mm, and leg length *l* = 7.5 mm, leads to *η* = 0.4%. For smaller dimensions than simulated here, the efficiency of this 4 leg module is expected to be 0.5%.

## Nomenclature

*A*: surface, m^2^
*a*: width, m
*b*: thickness, m
$\tilde{C}$: solar concentration ratio
*d*: distance between legs, m
e: charge of electron/charged particles, Coul
*F_k-j_*: view factor from surface *k* to *j*
g: gravitational acceleration, m s^-2^
*h*: heat transfer coefficient, W m^-2^ K^-1^
*j*: current density, A m^-2^
*k*: thermal conductivity, W m^-1^ K^-1^
*l*: leg length, m
*L*: absorber plate width, m
*N*: number of Monte Carlo rays
*P*: power output, W
*R*: resistance, Ω
*q^’’^*: heat flux, W m^-2^
$Q_{\text{solar}}$: incident solar power, W
$q_{\text{solar}}^{"}$: incident solar radiative flux, W m^-2^
*q^’’’^*: volumetric heat source, W m^-3^
*S*: Seebeck coefficient, V K^-1^
*t*: time, s
*T*: temperature, K
*V*: voltage, V
*v*: volume, m^3^
*x,y*: coordinates
Δ*V*: control volume, m^3^
Δ*A*: control surface, m^2^
Δ*x*: cell length in x-direction, m

## *Greek* *letters*

*α*: thermal diffusivity, m^2^ s^-1^
*β*: extinction coefficient, m^-1^
*β*: volumetric thermal expansion coeff, m^3^ K^-1^
*γ*: iteration step
*δ_kj_*: Kronecker delta
*ε*: total emissivity
*ε*: error
*η*: efficiency
*μ*: chemical potential, J mol^-1^
*ρ*: electrical resistivity, Ω m
*σ*: Stephan-Boltzmann constant, W m^-2^ K^-4^
ν: kinematic viscosity, m^2^ s^-1^
*ω*: scattering albedo

## *Subscripts*

*i, j*: finite volume indices
max: maximum
*m, n*: numbers of finite volumes
N: n-type
oc: open circuit
P: p-type
ray: single ray in Monte Carlo
∞: surroundings

## *Dimensionless* *group*

Gr: Grashof number
Nu: Nusselt number
Pr: Prandtl number
Ra: Rayleigh number
Re: Reynolds number
ZT: figure-of-merit

## *Abbreviations*

FV: Finite Volume
HFSS: high flux solar simulator
MC: Monte Carlo
N: n-type
OC: open circuit
P: p-type
PDE: Partial Differential Equation
SOR: successive over-relaxation
TEC: Thermoelectric converters
